# PECAM-1 Stabilizes Blood-Brain Barrier Integrity and Favors Paracellular T-Cell Diapedesis Across the Blood-Brain Barrier During Neuroinflammation

**DOI:** 10.3389/fimmu.2019.00711

**Published:** 2019-04-05

**Authors:** Isabella Wimmer, Silvia Tietz, Hideaki Nishihara, Urban Deutsch, Federica Sallusto, Fabien Gosselet, Ruth Lyck, William A. Muller, Hans Lassmann, Britta Engelhardt

**Affiliations:** ^1^Theodor Kocher Institute, University of Bern, Bern, Switzerland; ^2^Department of Neuroimmunology, Center for Brain Research, Medical University of Vienna, Vienna, Austria; ^3^Institute for Research in Biomedicine, Università della Svizzera italiana, Bellinzona, Switzerland; ^4^Institute of Microbiology, ETH Zürich,, Zurich, Switzerland; ^5^Blood-Brain Barrier Laboratory, Université d'Artois, Lens, France; ^6^Feinberg School of Medicine, Northwestern University, Chicago, IL, United States

**Keywords:** blood-brain barrier, endothelial junctions, PECAM-1, T-cell diapedesis, vascular permeability, multiple sclerosis

## Abstract

Breakdown of the blood-brain barrier (BBB) and increased immune cell trafficking into the central nervous system (CNS) are hallmarks of the pathogenesis of multiple sclerosis (MS). Platelet endothelial cell adhesion molecule-1 (PECAM-1; CD31) is expressed on cells of the vascular compartment and regulates vascular integrity and immune cell trafficking. Involvement of PECAM-1 in MS pathogenesis has been suggested by the detection of increased levels of soluble PECAM-1 (sPECAM-1) in the serum and CSF of MS patients. Here, we report profound upregulation of cell-bound PECAM-1 in initial (pre-phagocytic) white matter as well as active cortical gray matter MS lesions. Using a human *in vitro* BBB model we observed that PECAM-1 is not essential for the transmigration of human CD4^+^ T-cell subsets (Th1, Th1^*^, Th2, and Th17) across the BBB. Employing an additional *in vitro* BBB model based on primary mouse brain microvascular endothelial cells (pMBMECs) we show that the lack of endothelial PECAM-1 impairs BBB properties as shown by reduced transendothelial electrical resistance (TEER) and increases permeability for small molecular tracers. Investigating T-cell migration across the BBB under physiological flow by *in vitro* live cell imaging revealed that absence of PECAM-1 in pMBMECs did not influence arrest, polarization, and crawling of effector/memory CD4^+^ T cells on the pMBMECs. Absence of endothelial PECAM-1 also did not affect the number of T cells able to cross the pMBMEC monolayer under flow, but surprisingly favored transcellular over paracellular T-cell diapedesis. Taken together, our data demonstrate that PECAM-1 is critically involved in regulating BBB permeability and although not required for T-cell diapedesis itself, its presence or absence influences the cellular route of T-cell diapedesis across the BBB. Upregulated expression of cell-bound PECAM-1 in human MS lesions may thus reflect vascular repair mechanisms aiming to restore BBB integrity and paracellular T-cell migration across the BBB as it occurs during CNS immune surveillance.

## Introduction

Multiple sclerosis (MS) is a chronic demyelinating disorder of the central nervous system (CNS), which is characterized by a disease course-dependent degree of inflammation, axonal damage, oligodendrocyte death, and astrocytic scar formation affecting the white and gray matter of brain and spinal cord (1). Loss of blood-brain barrier (BBB) integrity is an early hallmark of acute and relapsing-remitting MS and can be visualized by the leakage of the magnetic resonance imaging (MRI) contrast agent gadolinium into the CNS parenchyma (2). T cells were shown to substantially contribute to MS pathogenesis (3, 4). Therapies targeting T-cell trafficking into the CNS by e.g., inhibiting T-cell egress from lymph nodes into the blood by fingolimod treatment or by restricting T-cell adhesion to the BBB endothelium by the humanized anti-α4 integrin antibody natalizumab, greatly reduce relapse rates in MS (5).

The BBB maintains CNS homeostasis, which is a prerequisite for the proper functioning of neurons and glial cells by shielding the CNS from an uncontrolled entry of blood-borne molecules. The highly specialized endothelial cells of the CNS microvasculature forming the BBB are part of the neurovascular unit, in which the interaction of pericytes, astrocytes as well as acellular extracellular matrix components ensure proper BBB function (6). Barrier characteristics of the highly specialized BBB endothelial cells are established by their low pinocytotic activity as well as their unique cell-to-cell junctions (6). In addition to regular adherens junctions established by the VE-cadherin/catenin complex, BBB endothelial cells are connected by complex and molecularly unique tight junctions established by members of the claudin and junctional adhesion molecule (JAM) families and the intracellular scaffolding proteins ZO-1, ZO-2, and ZO-3 [reviewed in (7)]. Outside of these organized junctional complexes, the BBB cell-to-cell contacts harbor additional transmembrane proteins such as platelet endothelial cell adhesion molecule-1 (PECAM-1) and CD99, which have been suggested to contribute to the regulation of vascular integrity and immune cell extravasation [reviewed in (7)].

Immune cell trafficking into the CNS is strictly controlled by the BBB and is a multistep process [reviewed in (8)]. It is initiated by a transient contact of the circulating immune cell with the BBB endothelium followed by rolling of the immune cell with reduced velocity along the vascular wall leading to its arrest, polarization, and subsequent crawling to sites permissive for diapedesis. Transmigration of immune cells across the vascular wall can either occur through the endothelial cell-cell junctions via the paracellular pathway mediated by the well-orchestrated rapid remodeling and sequential opening/closing of the interendothelial junctions in a zipper-like fashion (9), or through the endothelial cell body via a transcellular route by the formation a transcellular pore (10). It has long been thought that the unique cell-to-cell junctions of the BBB endothelium prohibit paracellular immune cell diapedesis across the BBB [reviewed in (11)]. However, recent evidence has shown that in the course of immune surveillance and thus in the absence of neuroinflammation, T cells preferentially cross the BBB via the paracellular route while transcellular T-cell penetration of the BBB is rather observed during active neuroinflammation as observed in experimental autoimmune encephalomyelitis (EAE), the animal model for MS (12, 13).

PECAM-1 is a type 1 transmembrane glycoprotein of the immunoglobulin (Ig) superfamily of cell adhesion molecules (14, 15). It contains six extracellular Ig-like domains and two cytoplasmic immune receptor tyrosine inhibitory motifs (16). PECAM-1 is expressed on platelets, endothelial cells, neutrophils, monocytes, and selected lymphocyte subsets (17–20). It is highly enriched at interendothelial junctions of vascular endothelial cells (17) and mediates neutrophil diapedesis across the vascular wall (21–23) as well as vascular integrity (24, 25). Moreover, it has been shown that PECAM-1 acts as vascular mechanosensor (26–28) and plays an important role in angiogenesis and vascular remodeling (29–32).

Involvement of PECAM-1 in MS pathogenesis is supported by our previous observation that PECAM-1^−/−^ C57BL/6 mice display aggravated clinical EAE due to impaired BBB integrity and accelerated immune cell infiltration into the CNS (24). In patients with relapsing-remitting MS, elevated serum levels of soluble PECAM-1 (33, 34), increased numbers of circulating PECAM-1-positive microparticles (35) as well as increased expression of PECAM-1 on circulating leukocytes (36) have been observed. Interestingly, interferon-β, a disease-modifying therapy for MS (37), increases vascular PECAM-1 expression (38) suggesting that an elevated expression of PECAM-1 might contribute to amelioration of MS.

Here, we found a profound upregulation of *PECAM-1* transcripts in initial (pre-phagocytic) white matter as well as active cortical gray matter MS lesions and localized the upregulated PECAM-1 protein to the vascular endothelium. We show that endothelial PECAM-1 contributes to the regulation of BBB integrity. Furthermore, while not required for the rate of T-cell diapedesis across the BBB, endothelial PECAM-1 was found to regulate the route of T-cell diapedesis, since its absence shifted T-cell migration across the BBB to the transcellular pathway. Our data suggest that increased vascular expression of PECAM-1 in MS may contribute to BBB stabilization and restoration of tightly controlled T-cell trafficking into the CNS.

## Materials and Methods

### RNA Isolation From FFPE Tissue and Whole-Genome Microarrays

Studies on human autopsy material were performed according to the Austrian legislation and were approved by the ethics committee of the Medical University of Vienna (No 535/2004). For the determination of *PECAM-1* transcription levels, pre-existing microarray data sets, which have already been published before with regard to other research questions (39–44), were once more re-evaluated. As described, well-characterized white and gray matter lesions from archival formalin-fixed paraffin-embedded (FFPE) autopsy tissue from MS patients (cases of acute MS for the dissection of white matter lesions; cases of secondary progressive MS for the dissection of gray matter lesions) as well as respective control tissue from controls cases without confounding neuropathology were dissected from multiple tissue sections. Overall, *PECAM-*1 expression in the periplaque white matter (PPWM) from 3 MS patients, 4 initial (pre-phagocytic) lesions (45, 46) from 3 MS patients and 5 active demyelinating lesions (47) from 4 MS patients was compared to transcript levels in normal white matter (NWM) from 4 controls. Likewise, active cortical gray matter lesions from 3 MS patients and normal cortical gray matter from 3 control cases were analyzed. Details on the study cohort, RNA isolation, quality control, microarray hybridization and data normalization have already been extensively described (39, 40).

### Immunohistochemistry

In total, we examined archival autopsy tissue of 20 MS patients including 6 cases of acute MS (death within 1 year after disease onset), 8 with secondary progressive MS and 6 with primary progressive MS. Additionally, 13 control cases without any confounding neuropathology were included. Characterization and classification of perilesional and lesional areas into different categories was done according to published criteria (48). Shortly described, the periplaque white matter is characterized by a loosened tissue structure due to edema and sparse perivascular inflammatory infiltrates and microglia activation can be detected, while myelin and oligodendrocytes are still intact. In initial (pre-phagocytic) lesions, myelin is still mostly preserved. Microglia activation, oligodendrocyte apoptosis and tissue edema are prominent, while tissue infiltration by peripheral macrophages is sparse. In active demyelinating white matter lesions, myelin is lost and myelin fragments are ingested by phagocytes, which numerously infiltrate these lesional areas. Likewise, tissue edema and dissolution of tissue structures is far advanced. Representative pictures for periplaque white matter, initial lesion and active demyelinating lesion were taken from an acute MS patients (34 years old; female; 4 months of disease duration); for active gray matter lesions from a secondary progressive MS case (42 years old; male; 216 months of disease duration); for normal white and gray matter areas from a 36 year-old female person. For antibody double-labeling of human FFPE tissue, 3–5 μm sections were routinely dewaxed in xylene and rehydrated in a descending ethanol series. Endogenous peroxidase activity was blocked by incubation of the slides in methanol/0.2% H_2_O_2_ for 30 min. For antigen retrieval, slides were steamed for 1 h in 10 mM Tris/1 mM EDTA buffer (pH 9.0). Thereafter, tissue sections were washed in Tris-buffered saline (TBS). Non-specific background binding was blocked with 10% fetal calf serum (FCS)/Dako buffer (Dako) for 20 min. Primary anti-PECAM-1 antibody (Neomarkers; diluted 1:200 in 10% FCS/Dako buffer) was applied and incubated over night at 4°C. Tissue sections were washed in TBS and incubated with biotinylated anti-mouse antibody (Jackson ImmunoResearch; diluted 1:500 in 10% FCS/Dako buffer) for 1 h at room temperature. After washing in TBS, PECAM-1 antibody labeling was developed with 3,3′-diaminobenzidine tetrahydrochloride hydrate (DAB). Subsequently, primary anti-PLP antibody (formerly provided by Prof. Paul Morgan, Cardiff University; diluted 1:250 in 10% FCS/Dako buffer) was applied and incubated over night at 4°C. Tissue sections were washed in TBS and incubated with an alkaline phosphatase-conjugated anti-rabbit antibody (Jackson ImmunoResearch; diluted 1:200 in 10% FCS/Dako buffer) for 1 h at room temperature. PLP staining was developed using the Fast Blue system as previously described (49). Tissue sections were mounted with geltol.

### Human T Cells

Human CD4^+^CD45RO^+^ T cells were sorted from the peripheral blood of healthy donors according to their specific expression pattern of chemokine receptors (CXCR3^+^ CCR4^−^ CCR6^−^ for Th1; CXCR3^+^ CCR4^−^ CCR6^+^ for Th1^*^; CXCR3^−^ CCR4^+^ CCR6^−^ for Th2; CXCR3^−^ CCR4^+^ CCR6^+^ for Th17) exactly as previously described (50, 51). T cells were cultured in the presence of interleukin-2 (IL-2; 500 U/ml) for a total of 20 days and were stored at −80°C. For each experimental run, T cells were thawed and dead cells were removed by a Ficoll gradient centrifugation (780 × g, 20 min, 20°C) and T cells were resuspended in migration assay medium (DMEM without phenol red/25 mM HEPES/5% fetal bovine serum (FBS)/4 mM L-glutamine). T cells were labeled with 1 μM CMFDA cell tracker (Life Technologies, ThermoFisher) for 30 min at 37°C.

### Human *in vitro* BBB Model and Transmigration Assay

The study protocol was approved by The French Ministry of Higher Education and Research (CODE-COH Number DC2011-1321) and written informed consent was obtained from the infants' parents prior to the collection of the infants' umbilical cord blood. The CD34^+^ cell-derived human *in vitro* BBB model was prepared exactly as described before (52, 53). Shortly described, brain-like endothelial cells (BLECs) were cultured on filter inserts (PC membrane, pore size 3.0 μm; Costar, 3402) for 7 days. Subsequently, they were co-cultured with bovine pericytes (52, 53) for 6 days to induce BBB-like characteristics. For the transmigration assay, BLECs were stimulated with both TNF-α (1 ng/ml; R&D Systems, 210-TA) and IFN-γ (20 IU/ml; R&D Systems, 285-IF) in the serum-containing complete Endothelial Cell Medium (ScienCell) for 16 h. Thereafter, BLECs were treated with either anti-human PECAM-1 (20 μg/ml; clone hec7), or anti-human CD99 (20 μg/ml; clone hec2) or anti-human ICAM-1 [10 μg/ml; clone BBIG-I1 (11C81), R&D Systems] antibodies, or the appropriate isotype controls for 30 min at 37°C. After incubation 1.5 × 10^5^ of the labeled T helper cells (either Th1, Th1^*^, Th2, or Th17 cells) were added to the upper chamber. T-cell transmigration was allowed for 8 h at 37°C in the presence of either blocking antibody or isotype control. The absolute numbers of transmigrated cells were counted using a CASY cell counter (OMNI Life Science).

### Mice

All mice were bred and housed in individually ventilated cages under specific pathogen-free conditions at the University of Bern. Experiments were carried out in compliance with the Swiss legislation on the protection of animals and the veterinary office of the Kanton of Bern (permission numbers: BE 66/12 and BE 72/15). All animals were from the C57BL/6 background. PECAM-1^−/−^ C57BL/6 mice were descendants from previously described PECAM-1 knock-out mice (54). VE-CadGFP knock-in mice (55) were kindly provided by D. Vestweber (Münster, Germany). PECAM-1^−/−^ VE-CadGFP mice were obtained by cross-breeding. Wild-type (WT) littermates were used as controls.

### Isolation of Primary Mouse Brain Microvasculature Endothelial Cells (pMBMECs)

Primary mouse brain microvasculature endothelial cells (pMBMECs) were isolated from 1 to 2 months old male and female mice and cultured exactly as described (56, 57). Capillaries from two brains allowed for seeding either 6 cloning rings (6 mm diameter), 4 filter inserts, 4 wells of a 16-well chamber slide, or 1 well of a 24-well plate. If isolated microvessels were not immediately plated, they were frozen in 100 μl freezing medium (30% fetal bovine serum, 10% DMSO, and 60% DMEM) per brain. After thawing, microvessels were seeded double as concentrated as freshly isolated ones.

### Mouse T Cells

Proteolipid protein (PLP)-specific CD4^+^ effector T cells from lines SJL.PLP4 and SJP.PLP7 were used 2–3 days after the 3rd, 4th, 5th, or 7th restimulation with their specific PLP peptide aa139-151 (58). Briefly, for the restimulation of established cell lines, spleens of SJL wild-type mice were collected, homogenized in Hank's balanced salt solution (HBSS)/25 mM HEPES buffer/5% calf serum using a sterile Wheaton^TM^ homogenizer (Loose pestle), filtered (100 μm mesh size) and irradiated with 40 Gy (Gammacell 3000 Elan, Nordion International Inc.). Afterwards, red blood cells were lysed. Generally, 1.5–2 × 10^6^ resting T cells were restimulated with 3–4 × 10^6^ splenocytes and 10 μg/ml PLP peptide in 60 mm dishes filled with 5 ml restimulation medium (RPMI, 10% FBS, 4 mM L-glutamine, 100 U/ml penicillin, 100 μg/ml streptomycin, 1% non-essential amino acids, 1 mM sodium pyruvate, 50 μM β-mercaptoethanol). After 48 h incubation (37°C and 7% CO_2_), 1 ml TCGF medium (restimulation medium supplemented with IL-2-containing cell supernatant) was added to each dish. After 16 h, activated T-cell blasts were purified on a NycoPrep^TM^ 1.077 gradient (Progen Biotechnik GmbH) and seeded at a density of 2.5–3 × 10^6^ cells per 100 mm dish in TCGF medium. The antigen-specific activated T cells were either used for experiments or cultured for 9–11 days and subsequently subjected to another round of restimulation.

### *In vitro* Live Cell Imaging Under Flow Conditions

WT and PECAM-1^−/−^ pMBMECs were seeded in cloning rings placed on matrigel-coated Ibidi μ-dishes. Six to seven days after isolation, pMBMEC monolayers were stimulated with 20 ng/ml IL-1β (PeproTech) in the presence of serum and flow movies were recorded within 16–24 h post stimulation. Generally, *in vitro* live cell imaging was done as described (59). Briefly, a custom-made flow chamber was mounted on an Ibidi dish, placed on a heated stage of an inverted microscope (AxioObserver Z1 equipped with a monochrome CCD camera; both Zeiss) and connected to an automated syringe pump (Harvard Apparatus). Using low shear stress (0.1 dyn/cm^2^), T cells were perfused over the pMBMEC monolayer and allowed to accumulate for 5 min. Thereafter, medium flow was increased to physiological shear stress (1.5 dyn/cm^2^). To study the interaction of T cells with pMBMECs, 1 × 10^6^ T cells/ml (DMEM/2% L-glutamine/25 mM HEPES/5% calf serum) were perfused over the endothelial monolayer and phase-contrast images were acquired every 5 s for a total of 20 min using a 20 × objective. For the analysis of time-lapse videos, ImageJ software (National Institutes of Health) was used. First, the number of arrested T cells was counted one frame after increasing the flow rate to physiological shear. Then, the behavior of each arrested T cell was determined and classified into 4 categories as follows: T cells either detached during the observation period (“detachment”), remained situated at the same position mostly probing their surrounding via their protrusions (“probing”), continuously crawled on the endothelial surface (“crawling”), or migrated across the pMBMEC monolayer (with or without prior crawling; “diapedesis”). If T cells exhibited any moving behavior, defined as cell displacement of more than one cell diameter, the crawling path was tracked using the Manual Tracking plugin of ImageJ (https://imagej.nih.gov/ij/plugins/track/track.html). Crawling speed, crawling distance and crawling directionality of each T cell were calculated using the Chemotaxis and Migration Tool plugin (https://ibidi.com/chemotaxis-analysis/171-chemotaxis-and-migration-tool.html). To determine the diapedesis route, 2 × 10^6^ T cells/ml (DMEM without phenol red/2% L-glutamine/25 mM HEPES/5% calf serum) were perfused over VE-CadGFP pMBMECs (WT as well as PECAM-1^−/−^) and differential interference contrast (DIC) pictures as well as pictures with a GFP filter were taken every 30 s for a total of 30 min with a 63 × objective. T-cell diapedesis interrupting the continuous junctional VE-CadGFP signal was categorized as paracellular diapedesis. Diapedesis, which did not interrupt the continuous junctional GFP signals, was classified as transcellular. Each video represents an independent experiment carried out with separate pMBMECs and T-cell aliquots.

### Immunofluorescence Stainings

WT and PECAM-1^−/−^ pMBMECs were seeded on matrigel-coated 16-well glass Nunc™ Lab-Tek™ chamber slides (Thermo Fisher Scientific) or removable 12-well chamber glass slides (Ibidi). If required, pMBMECs were stimulated at the 6th day after isolation with IL-1β (20 ng/ml) in the presence of serum for 16 h. Seven days after isolation, cells were fixed with 100 μl 1% PFA for 10 min or, in the case of occludin and VE-cadherin, in −20°C-cold methanol for 30 s. Thereafter, cells were permeabilized in blocking buffer (5% skimmed milk, 0.3% Triton X-100, 0.04% NaN_3_ in TBS) for 20 min. Hybridoma supernatants [rat-anti-mouse: PECAM-1 (Mec13.3), VE-cadherin (11D4), ICAM-1 (25ZC7), JAM-A (BV12); rat-anti-human CD44 (9B5) used as isotype control] were applied without prior dilution. ZO-1 antibody (rabbit polyclonal; 1:100; Thermo Fisher Scientific), rabbit polyclonal occludin antibody (1.25 μg/ml Thermo Fisher Scientific), rabbit polyclonal claudin-5 antibody (1.25 μg/ml, Thermo Fisher Scientific) and rabbit IgG (R&D Systems) were diluted in blocking solution. Primary antibodies were incubated for 30 min at room temperature. After washing of wells with PBS, secondary antibodies (goat-anti-rat Alexa488, Thermo Fisher Scientific; goat-anti-rabbit Cy3, Jackson ImmunoResearch) were applied together with DAPI (0.5 μg/ml) for 30 min at room temperature and slides were mounted in Mowiol.

### Western Blot

WT and PECAM-1^−/−^ pMBMECs were seeded in matrigel-coated 24-well plates. Six days after isolation, pMBMEC monolayers were stimulated or not with IL-1β (20 ng/ml) for 16 h. Cells were washed and lysed in 70 μl ice-cold RIPA buffer [50 mM Tris, 150 mM NaCl, 0.1% sodium deoxycholate, 0.1% sodium dodecyl sulfate, 1% NP-40, protease inhibitors (Roche), pH 7.5]. Protein quantity was determined using the Pierce^TM^ BCA Protein Assay Kit according to manufacturer's instructions. Hundred microgram of each sample were mixed with 2X SDS sample buffer, heated to 95°C for 5 min and separated by SDS-PAGE (10% resolving gel; 90 V for 30 min and 120 V for 1.5 h). Proteins were transferred to a nitrocellulose membrane (semi-dry blotting at 15 V for 15 min), which was blocked for 1 h in 5% skimmed milk powder in TBST buffer. Polyclonal rabbit anti-ICAM-1 raised against mouse immune globulin domains 1 and 2 (custom made, Eurogentec, Seraing, Belgium) (53) was diluted 1:1000 in 5% skimmed milk powder/TBST and incubated overnight at 4°C. After washing with TBST, GAPDH antibody (rabbit monoclonal; Cell Signaling Technology; 1:1000) was applied for 1 h at room temperature. Both antibodies were visualized simultaneously with IRDye680-conjugated goat-anti-rabbit IgG (Li-Cor Biosciences) incubated for 1 h at room temperature. Signals were visualized using an Odyssey reader (Li-Cor Biosciences) and quantified via Image Studio Lite v4.0 (Li-Cor Biosciences). Background subtraction was done automatically. Protein size was referenced to a pre-stained protein ladder (Thermo Fisher Scientific, 26635). For each sample, ICAM-1 signals were normalized to GAPDH. Furthermore, IL-1β-stimulated WT and PECAM-1^−/−^ as well as untreated PECAM-1^−/−^ samples were further related to untreated WT samples. Reported results derive from four independent experiments.

### Impedance Spectroscopy

WT and PECAM-1^−/−^ pMBMECs were seeded on laminin- and matrigel-coated filter inserts (transparent PET membrane, pore size 0.4 μm; Greiner Bio-one, 662641). On the following 2 days, cells were thoroughly washed during medium change. Then, 48 h after cell isolation, filter inserts were transferred to the cellZscope® (nanoAnalytics) for impedance spectroscopy allowing to monitor the transendothelial electrical resistance (TEER; one measurement per hour) over several days. After a stable TEER plateau had formed, pMBMECs were either treated with IL-1β (final concentration: 20 ng/ml) or the identical volume of PBS. The experiment was terminated 2 days later. Via the cellZscope® software, hourly TEER values for each filter insert were exported to Microsoft Excel. To determine TEER differences between WT and PECAM-1^−/−^ pMBMECs during the stable plateau phase, the maximal resistance values 80–90 h after initiating cellZscope measurements were analyzed and reported in relation to WT cells. In order to analyze the effect of IL-1β stimulation, the ratio between the TEER value 16 h after cytokine/PBS treatment and the TEER value immediately before stimulation was calculated for each filter insert. Thereafter, ratios of IL-1b-treated WT and PECAM-1^−/−^ samples were related to ratios of the respective controls.

### Permeability Assays

WT and PECAM-1^−/−^ pMBMECs were seeded on laminin- and matrigel-coated filter inserts (translucent polycarbonate membrane, pore size 0.4 μm; Corning Costar). Six days after isolation, pMBMEC monolayers were stimulated with IL-1β (20 ng/ml)- or PBS-conditioned medium for 16 h. Permeability assays and permeability coefficient (Pe) calculations were performed as described previously (56). Shortly described, 50 μM Lucifer Yellow CH dilithium salt (Sigma-Aldrich) and 10 μg/ml Alexa–Fluor-680-conjugated 3 kDa dextran (Molecular Probes) were loaded into the upper compartment of the filter inserts. For the clearance experiments, the amount of fluorescent tracer diffusing through the endothelial monolayer was determined every 10 min for a total of 40 min and quantified using an Infinite M1000 (Tecan) and Odyssey (Li-Cor Biosciences) reader for Lucifer yellow and dextran, respectively. Permeability coefficients were calculated for each insert according to Coisne et al. (56). Assays were carried out in HBSS without phenol red supplemented with 5% fetal bovine serum and 25 mM HEPES. Each experiment was done in technical triplicates. Analyses combine four independent experiments.

### Determination of Monolayer Integrity

After termination of permeability assays and TEER measurements, filter inserts were fixed in 3.7% formaldehyde for 10 min and incubated for 20 min in staining solution (PBS, 1% BSA, 0.1% Triton X-100, 12.5 μg/ml rhodamine phalloidin, 0.5 μg/ml DAPI). Filters were cut out and mounted. Integrity of pMBMEC monolayers was determined at the AxioObserver Z1 (equipped with Cy3 and Dapi filters) using ZEN 2012 blue edition software (both Carl Zeiss).

### Statistics

For statistical analysis, GraphPad Prism v.6.01 was used. Generally, reported statistics result from unpaired, two-tailed Student's *t*-tests, one-way ANOVA with either Dunnett's or Sidak's multiple comparisons test or from Pearson's qui-square test. Microarray data were analyzed by non-parametric Kruskal-Wallis and Mann-Whitney U-tests. Asterisks indicate significant differences (^*^p < 0.05, ^**^p < 0.01, ^***^p < 0.001).

## Results

### PECAM-1 Expression Is Increased in Initial White Matter and Active Cortical MS Lesions

**Figure 1:**
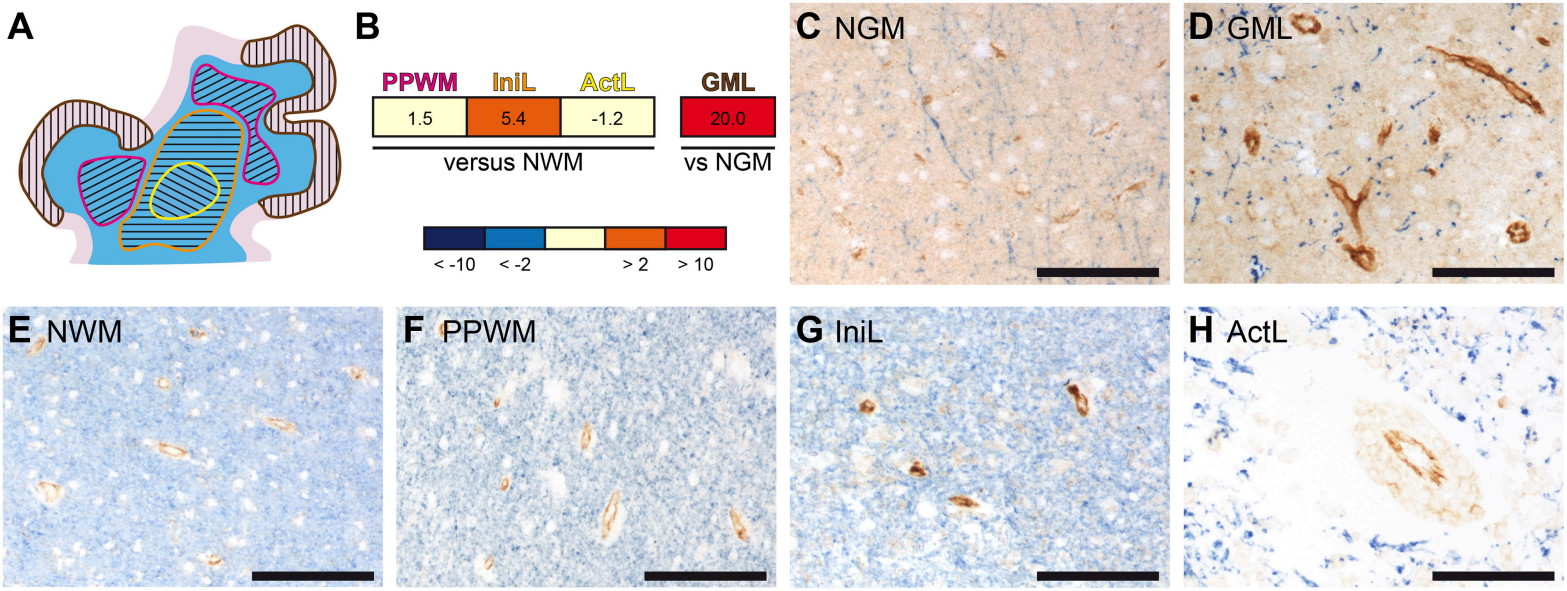
Gene and protein expression of PECAM-1 is pronounced in initial white matter MS lesions and active cortical MS lesions. **(A)** Representative schematic overview of the different MS lesions included in the microarray analyses. The separately microdissected zones are encircled by differently colored lines: yellow, active demyelinating lesion; orange, initial (pre-phagocytic) lesion; pink, periplaque white matter; brown, active cortical gray matter lesion. White matter is depicted in blue; the cortical ribbon is depicted in rose. **(B)** Gene expression analysis in microdissected MS brain lesions. *PECAM-1* expression in affected white matter areas (*n* = 4 patients) was compared to normal white matter of healthy controls (*n* = 4 subjects); *PECAM-1* expression in active cortical gray matter MS lesions (*n* = 3 patients) was compared to cortical areas isolated from healthy controls (*n* = 3 subjects). Non-parametric analyses did not reach any significance levels (white matter: *H*(3) = 1.888, *p* = 0.6284; gray matter: *U* = 0.000, *z* = −1.964, *p* = 0.100, *r* = −0.802). PPWM, periplaque white matter; IniL, initial (pre-phagocytic) lesions; ActL, active demyelinating lesions; NWM, normal white matter; GML: gray matter lesion; NGM: normal gray matter **(C–H)** Immunohistochemical double-labeling for PECAM-1 (brown) and the myelin component proteolipid protein (PLP; blue) of normal gray matter of control **(C)**, active cortical gray matter MS lesion **(D)**, normal white matter of control **(E)**, periplaque white matter **(F)**, initial (pre-active) MS lesions **(G)** and active MS lesions **(H)**. Representative pictures are shown. Scale bars, 100 μm.

In previous studies, we have performed gene expression analyses comparing active cortical MS lesions to cortical tissue of other inflammatory and neurodegenerative brain diseases as well as different stages in white matter lesion development to normal white matter of controls. We identified potential molecular pathways of tissue injury and reported on the differential expression of oxidative stress-, microglia-, and iron-related genes (39–44). In addition these microarray data sets revealed a pronounced upregulation of *PECAM-1* transcripts in MS lesions (Figures 1A,B). In comparison to normal white matter (NWM) from non-neurological controls, *PECAM-1* expression was more than 5-fold increased in initial (pre-phagocytic) white matter MS lesions (IniL), while it was only slightly upregulated in the periplaque white matter (PPWM) and unchanged in active demyelinating lesions (ActL). In active cortical gray matter lesions (GML), we noticed a pronounced expression of *PECAM-1* in comparison to cortical tissue from control patients (NGM). Immunohistochemical labeling confirmed the differential expression of PECAM-1 during MS lesion maturation on vascular endothelial cells. Endothelial PECAM-1 staining was most pronounced in initial (pre-phagocytic) MS lesions (Figure 1G), while it was less strong in the PPWM (Figure 1F), active demyelinating lesions (Figure 1H) or the NWM of controls (Figure 1E). Likewise, PECAM-1 expression by endothelial cells was higher in cortical gray matter MS lesions (Figure 1D) compared to cortical tissue of control cases without confounding neuropathology (Figure 1C). Based on our results it is tempting to speculate that dysregulated endothelial PECAM-1 expression may influence MS pathogenesis.

### Endothelial PECAM-1 Is Not Essential for T-Cell Diapedesis Across a Static Human *in vitro* BBB Model

**Figure 2:**
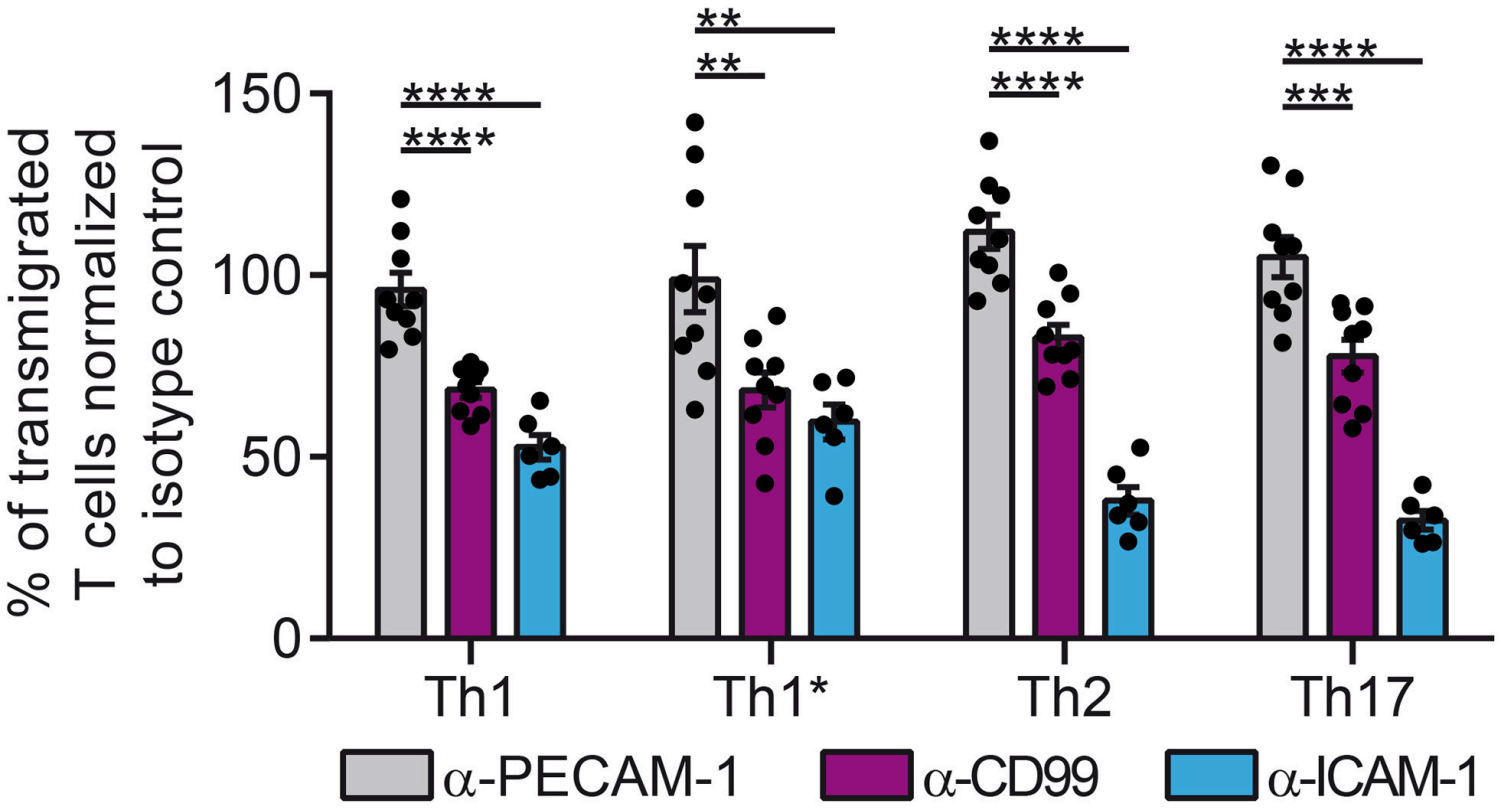
PECAM-1 is not required for the diapedesis of human effector/memory CD4^+^ T cells across BLECs. Diapedesis of human CD4^+^ T-cell subsets (Th1, Th1^*^, Th2, and Th17) across the TNF-α-(1 ng/ml) + IFN-γ-stimulated (20 IU/ml) human *in vitro* BBB model (BLECs) is shown. Prior to addition of T cells, BLECs were pre-treated with anti-human PECAM-1, anti-human CD99 or anti-human ICAM-1 blocking antibodies or the adequate isotype control antibodies. T cells were allowed to migrate for 8 h across the BLEC monolayers. Transmigration rates are expressed relative to isotype control-treated filter inserts (100%). Data are derived from three independent experiments each performed in triplicates, except for ICAM-1 blocking conditions, which were done in two independent experiments each performed in triplicates. T cells were derived from 3 different healthy donors. Dots represent individual data points; error bar ± SEM. Reported statistics result from one-way ANOVAs with Dunnett's multiple comparisons tests. ^**^p < 0.01; ^***^p < 0.001; ^****^p < 0.0001.

We first asked if a dysregulated vascular PECAM-1 expression would influence T-cell trafficking into the CNS. To this end, we tested if PECAM-1 is involved in the migration of human effector/memory CD4^+^ T cells across a human *in vitro* model of the BBB (52, 53). Using static conditions and 4 subsets of human T helper cells [classical Th1, Th1^*^,Th2, Th17 (60)] we found that functional antibody-mediated blockade of endothelial PECAM-1 does not interfere with the transmigration rate of the different T-cell subsets of human effector/memory CD4^+^ T cells across our TNF-α-/IFN-γ-stimulated human BBB model (Figure 2), although a small percentage of Th1 and Th2 cells expressed PECAM-1 allowing for homophilic interactions (Supplementary Figure 1). At the same time, addition of anti-human CD99 or anti-human ICAM-1 blocking antibodies greatly reduced the rate of T-cell diapedesis and confirmed the validity of our experimental setup. Thus, PECAM-1 is not required for the diapedesis of human effector/memory CD4^+^ T cells across our human *in vitro* BBB model.

### PECAM-1^−/−^ pMBMECs Maintain an Intact Junctional Architecture

**Figure 3:**
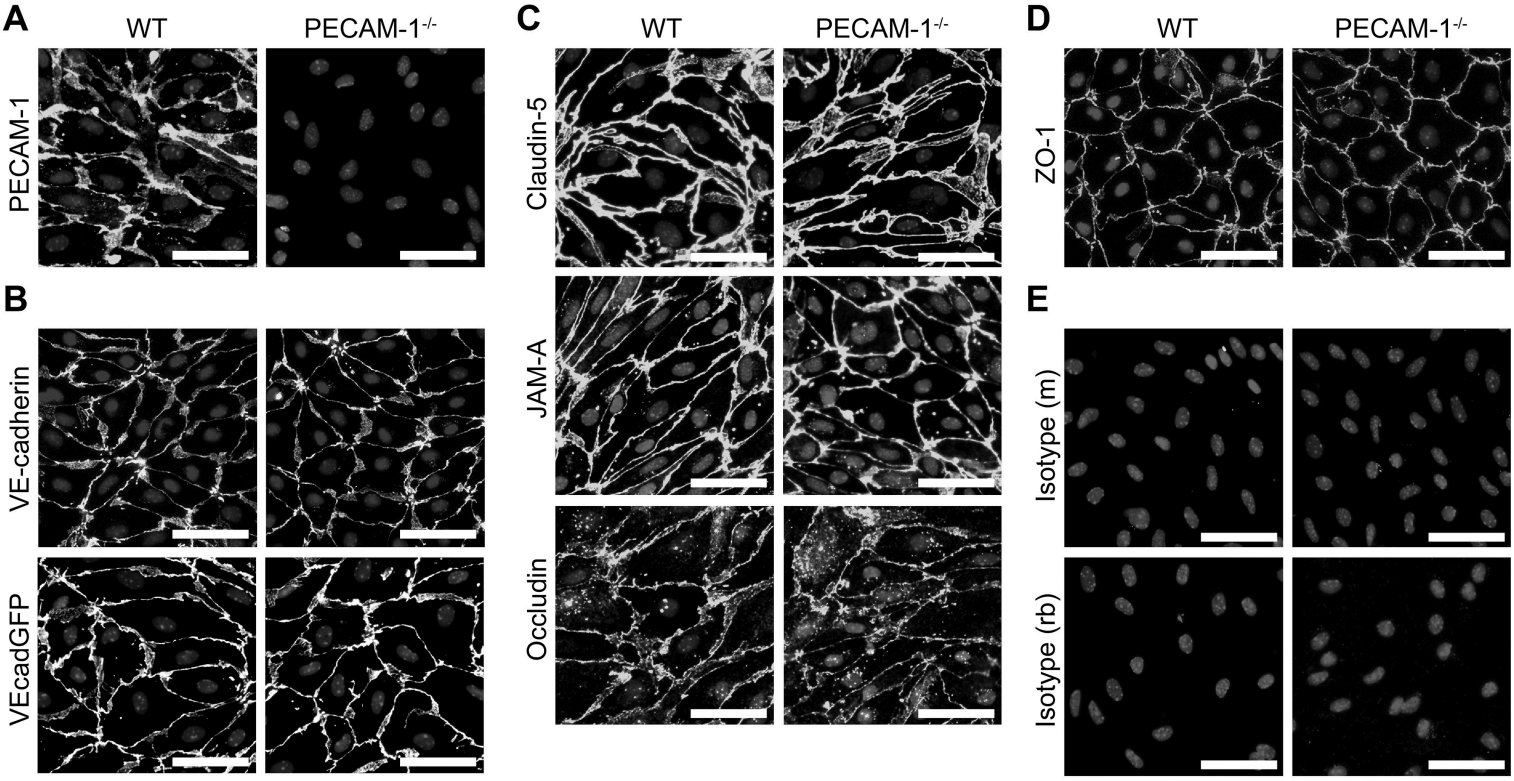
PECAM-1^−/−^ pMBMECs display an intact junctional architecture. Immunofluorescent stainings of WT and PECAM-1^−/−^ pMBMEC monolayers were performed for PECAM-1 **(A)**, VE-cadherin **(B)**, claudin-5 **(C)**, JAM-A **(C)**, occludin **(C)**, and ZO-1 **(D)**. Additionally, the fluorescent signal of VE-CadGFP-expressing pMBMECs is shown **(B)**. Also, stainings for mouse and rabbit isotype controls are presented **(E)**. Cell nuclei were stained with DAPI. Merged grayscale pictures of nucleic signals and the specific antibody stainings are shown. Scale bars, 50 μm.

Next we asked if dysregulated endothelial expression of PECAM-1 may also lead to BBB dysfunction. To test this hypothesis, we employed our well-established mouse *in vitro* model for the BBB (56), in which primary mouse brain microvascular endothelial cells (pMBMECs) form a tight and polarized cellular barrier (61). First, we generated pMBMEC cultures from wild-type (WT) and PECAM-1^−/−^ C57BL/6 mice and further investigated if lack of PECAM-1 affects the basic barrier architecture of pMBMEC monolayers. Both WT and PECAM-1^−/−^ pMBMECs formed confluent monolayers. Junctional immunostaining for PECAM-1 was prominent in WT, but absent in PECAM-1^−/−^ pMBMECs (Figure 3A). WT and PECAM-1^−/−^ pMBMECs displayed indistinguishable junctional localization of the transmembrane adherens junction molecule VE-cadherin (Figure 3B) as well the transmembrane tight junction proteins claudin-5, JAM-A and occludin (Figure 3C). Also, the junctional localization of the intracellular scaffolding protein zonula occludens-1 (ZO-1) was unaffected by the lack of PECAM-1 (Figure 3D). Isotype control stainings did not show differences between WT and PECAM-1^−/−^ pMBMECs (Figure 3E). Thus, the overall molecular architecture of interendothelial junctions was not affected by the absence of PECAM-1.

### Lack of Endothelial PECAM-1 Impairs Barrier Integrity of pMBMEC Monolayers

**Figure 4:**
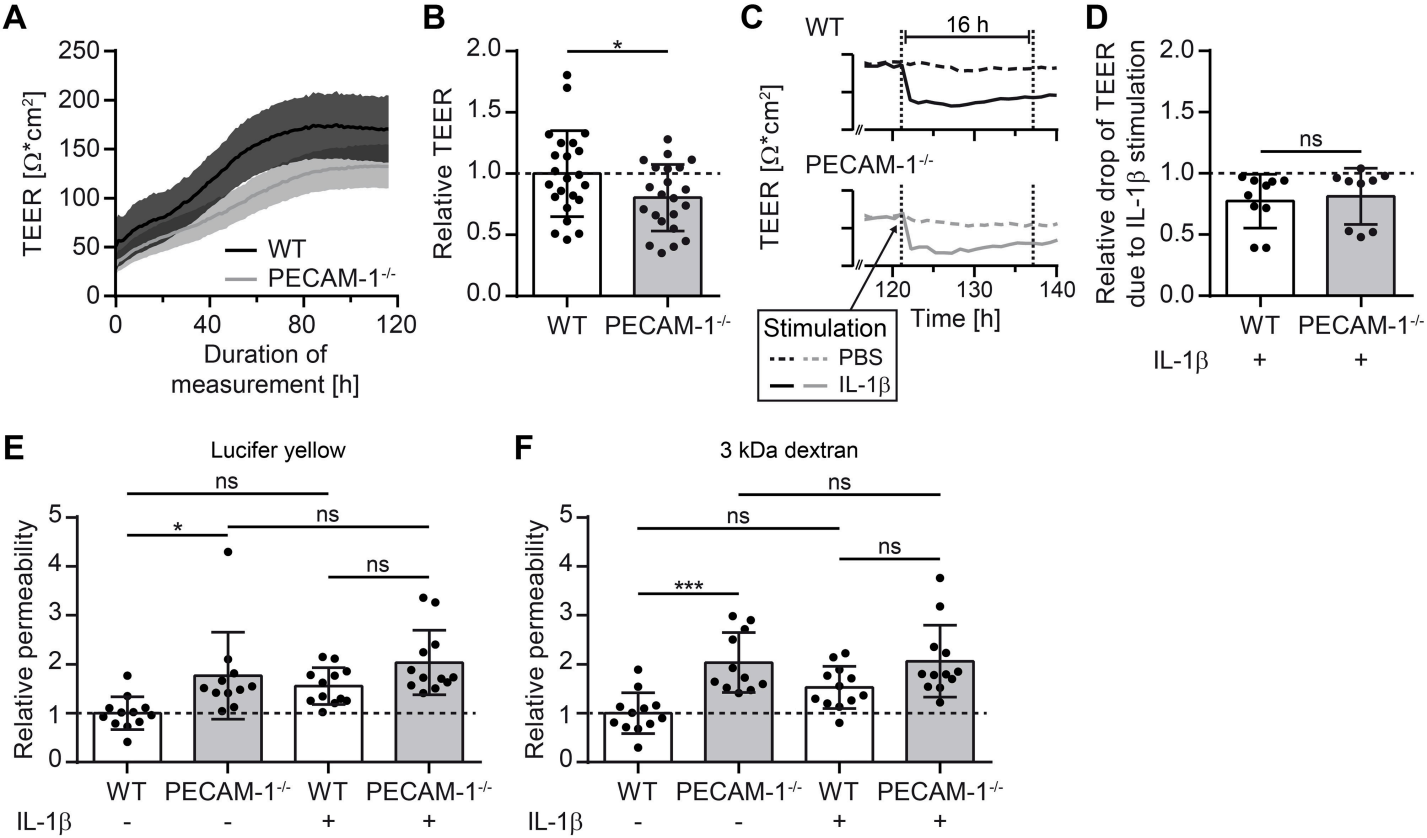
PECAM-1^−/−^ pMBMECs exhibit impaired barrier properties. **(A)** Transendothelial electrical resistance (TEER) of pMBMECs grown on filter inserts was measured by impedance spectroscopy using the cellZscope® (nanoAnalytics) starting 2 days after cell isolation over a period of 5 days in hourly intervals. Solid lines (black, WT; gray, PECAM-1^−/−^) represent mean values and shaded areas represent SEM calculated from 6 filter inserts with WT pMBMECs and 5 filter inserts with PECAM-1^−/−^ pMBMECs, respectively. One representative experiment out of three is shown. **(B)** Relative TEER of WT and PECAM-1^−/−^ pMBMECs was calculated from maximal TEER of each filter insert with WT set to 1.0. Graph combines three independent experiments (WT, 23 filter inserts; PECAM-1^−/−^, 22 filter inserts). Dots represent individual data points; error bar ± SD. Reported statistics result from unpaired, two-tailed Student's *t*-test. Test statistics: *p* = 0.042, *t*(43) = 2.096, *d* = 0.639. **(C)** Exemplary TEER curves for PBS- and IL-1β-treated WT and PECAM-1^−/−^ pMBMEC monolayers are shown. Dashed lines, PBS-treated filter inserts; solid lines, IL-1β-treated filter inserts; dotted vertical lines, 16 h time window of stimulation. **(D)** Relative drop of TEER indicating the decrease of electrical resistance 16 h after stimulation (final concentration of 20 ng/ml IL-1β per insert) normalized to genotype-matched PBS-treated filter inserts. Dots represent individual data points; error bar ± SD. Reported statistics result from unpaired, two-tailed Student's *t*-test. Test statistics: *p* = 0.714, *t*(17) = 0.373, *d* = 0.181. **(E,F)** Relative permeability coefficient of the two different sized fluorescent tracers Lucifer yellow (457 Da) **(E)** and Alexa-Fluor-680-conjugated 3 kDa dextran **(F)** across WT and PECAM-1^−/−^ pMBMEC monolayers normalized to the mean WT value. Data are derived from four independent experiments (WT, 12 filter inserts; PECAM-1^−/−^, 11 to 12 filter inserts). Dots represent individual data points; error bar ± SD. Reported statistics result from one-way ANOVAs with Sidak's multiple comparisons test. Test statistics: **(E)** *F*(3, 43) = 6.382, *p* = 0.0011, η^2^ = 0.308; **(F)** *F*(3, 43) = 9.375, *p* < 0.0001, η^2^ = 0.395; ^*^p < 0.05; ^***^p < 0.001; ns, not significant.

As a next step, we investigated barrier characteristics of pMBMEC monolayers established from WT and PECAM-1^−/−^ C57BL/6 mice. First, we examined the transendothelial electrical resistance (TEER) of the pMBMEC monolayers by impedance spectroscopy starting 48 h after seeding the freshly isolated brain capillaries. TEER values increased comparably for both WT and PECAM-1^−/−^ pMBMECs reaching a plateau with a maximum TEER at day 5 (approximately 72 h after start of measurements) that subsequently remained constant for several days (Figure 4A). Lack of endothelial PECAM-1 did not delay barrier formation of pMBMEC monolayers, however, the maximum TEER of PECAM-1^−/−^ pMBMECs was significantly lower compared to WT pMBMECs (Figure 4B). Next, we asked, whether these already reduced barrier properties of PECAM-1^−/−^ pMBMECs would be further affected under inflammatory conditions. We have previously reported that the pro-inflammatory cytokine IL-1β impairs barrier characteristics of pMBMEC monolayers (12). In agreement with these observations, IL-1β stimulation of WT pMBMECs reduced the TEER of the monolayers when compared to control treatment (Figure 4C). For PECAM-1^−/−^ pMBMECs, which displayed already a significantly lower TEER under unstimulated conditions (Figure 4B), IL-1β treatment further reduced the TEER to the same degree as observed for WT pMBMECs (Figure 4D). Thus, IL-1β reduced the TEER across WT and PECAM-1^−/−^ pMBMEC monolayers in a comparable and thus PECAM-1-independent manner.

Barrier properties of confluent pMBMEC monolayers were further investigated by examining their permeability for the two fluorescent tracers Lucifer yellow (457 Da) and Alexa-Fluor-680-conjugated 3 kDa dextran. Both tracers diffused at a significantly higher rate across unstimulated PECAM-1^−/−^ pMBMEC monolayers when compared to unstimulated WT pMBMECs (Figures 4E,F). IL-1β treatment was previously found to elevate the diffusion rates of small molecular tracers across pMBMECs (12). Interestingly, upon IL-1β stimulation, PECAM-1^−/−^ pMBMECs did not exhibit a further increase in permeability for Lucifer Yellow or Alexa-Fluor-680-conjugated 3 kDa dextran, when compared to IL-1β-stimulated WT pMBMECs (Figures 4E,F). This may be due to the fact that the barrier function of unstimulated PECAM-1^−/−^ pMBMECs is already too impaired, thus masking a further increase of tracer diffusion after stimulation with IL-1β in this experimental setup. Nevertheless, these observations underscore the involvement of PECAM-1 in regulating BBB integrity. Taken together, absence of PECAM-1 significantly impaired barrier properties of pMBMEC monolayers without visibly affecting the overall molecular architecture of their adherens and tight junctions.

### Absence of Endothelial PECAM-1 Does Not Affect Arrest and Crawling of Encephalitogenic T Cells on pMBMECs

**Figure 5:**
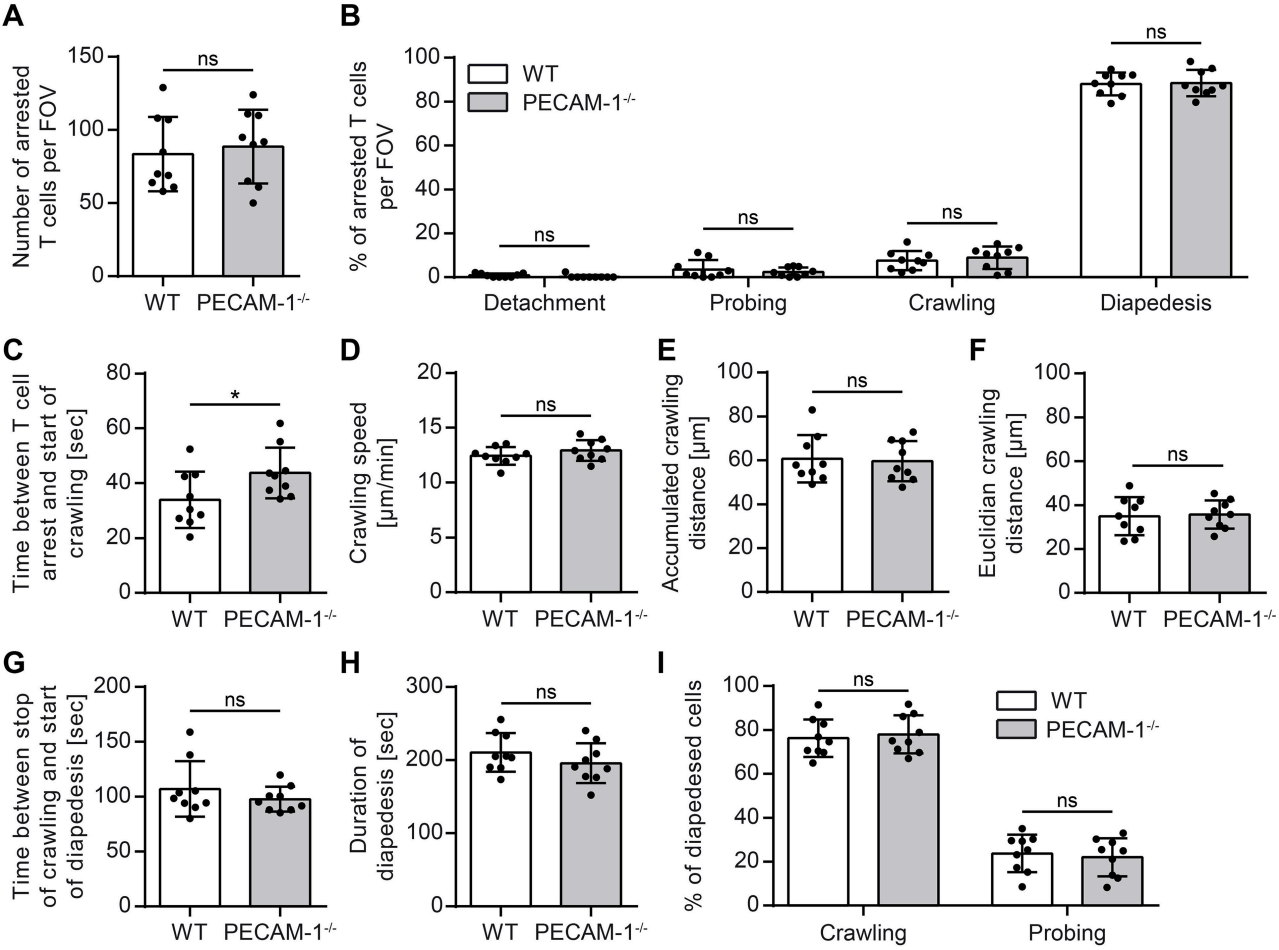
Lack of endothelial PECAM-1 does not affect T-cell arrest or post-arrest T-cell crawling and diapedesis. Confluent WT and PECAM-1^−/−^ pMBMEC monolayers were stimulated with IL-1β (20 ng/ml) for 16–24 h prior to live cell imaging. Activated encephalitogenic CD4^+^ T cells were allowed to arrest on pMBMECs at low flow (0. 1 dyn/cm^2^). After 5 min, the flow was increased to 1.5 dyn/cm^2^. For time-lapse videos, pictures were taken every 5 s for a total of 20 min. **(A)** The number of arrested T cells after increasing the flow rate (frame 60). **(B)** The behavior of each arrested T cell was determined for the subsequent 15 min (frame 60 to 240) and assigned to one of the following categories: detachment, probing behavior, continuous crawling, diapedesis. **(C)** For all T cells eventually starting to crawl, the time span between initial arrest to the pMBMECs and the first frame of crawling was determined. **(D–F)** Each T cell crawling on the activated endothelial monolayer was tracked and the crawling speed **(D)**, accumulated crawling distance **(E)**, and Euclidian crawling distance **(F)** were calculated. **(G)** For all crawling T cells eventually starting to transmigrate, the time span between halt of movement and start of diapedesis was calculated. **(H)** For all T cells that finished diapedesis, the duration of diapedesis was calculated. **(I)** T cells, which eventually started to transmigrate, were classified according to their prior behavior: crawling or stationary. Data are derived from 9 videos per pMBMEC genotype performed in three independent experiments. Error bar, ± SD; FOV, field of view; ns, not significant. Reported statistics result from unpaired, two-tailed Student's *t*-tests. Test statistics for (C): *p* = 0.0488, *t*(16) = 2.132 (*d* = 1.066); ^*^p < 0.05; ns, not significant.

We and others have previously shown that increased vascular permeability does not allow predicting effects on immune cell trafficking across the vascular border (12, 62). On the other hand we had previously observed that PECAM-1^−/−^ C57BL/6 mice develop early onset of EAE due to enhanced immune cell trafficking into the CNS (24). Therefore, to address this, we studied the interaction of effector/memory CD4^+^ T cells with pMBMEC monolayers under physiological flow by *in vitro* live cell imaging (59). The employed encephalitogenic T cells expressed PECAM-1 allowing for homophilic interaction with endothelial PECAM-1 (Supplementary Figure 1). The behavior of those T cells that arrested on the pMBMEC monolayers under physiological shear forces was classified into 4 categories (detachment, probing, crawling, and diapedesis) as described in the Materials and Methods section. We found that encephalitogenic CD4^+^ T cells arrested in comparable numbers on IL-1β-treated WT and PECAM-1^−/−^ pMBMECs (Figure 5A). From this pool of arrested cells, a minority constantly crawled over IL-1β-stimulated WT and PECAM-1^−/−^ pMBMEC monolayers during an observation period of 20 min, while the majority underwent diapedesis irrespective of previous crawling (Figure 5B). A small, comparable percentage of arrested T cells remained probing on both WT and PECAM-1^−/−^ pMBMECs, while a negligible, and again comparable, number of T cells detached from both WT and PECAM-1^−/−^ pMBMEC monolayers during the observation time. Taken together, absence of endothelial PECAM-1 did not affect any post-arrest T-cell behavior on IL-1β-stimulated pMBMEC monolayers under flow *in vitro* (Figure 5B).

Interestingly, T cells that eventually crawled on the pMBMEC monolayers needed significantly more time to start their active crawling behavior on PECAM-1^−/−^ pMBMECs compared to WT pMBMECs (Figure 5C). Other T-cell parameters such as crawling speed, accumulated crawling distance and Euclidian crawling distance on IL-1β-stimulated pMBMECs were not influenced by the lack of endothelial PECAM-1 (Figures 5D–F). Also, the time lag between the stop of T-cell crawling and start of diapedesis across IL-1β-stimulated pMBMEC monolayers (Figure 5G), the overall duration of T-cell diapedesis (Figure 5H) as well as the percentages of T cells, which crawled or stayed stationary prior to diapedesis (Figure 5I) were not altered between WT and PECAM-1^−/−^ pMBMECs.

Altogether, we found that the absence of endothelial PECAM-1 hardly affected common parameters of T-cell interaction with IL-1β-stimulated pMBMECs. Solely the time span, which T cells needed to start post-arrest crawling, was significantly influenced by the lack of endothelial PECAM-1.

### Lack of Endothelial PECAM-1 Favors Transcellular Over Paracellular T-Cell Diapedesis Across pMBMEC Monolayers Under Physiological Flow

**Figure 6:**
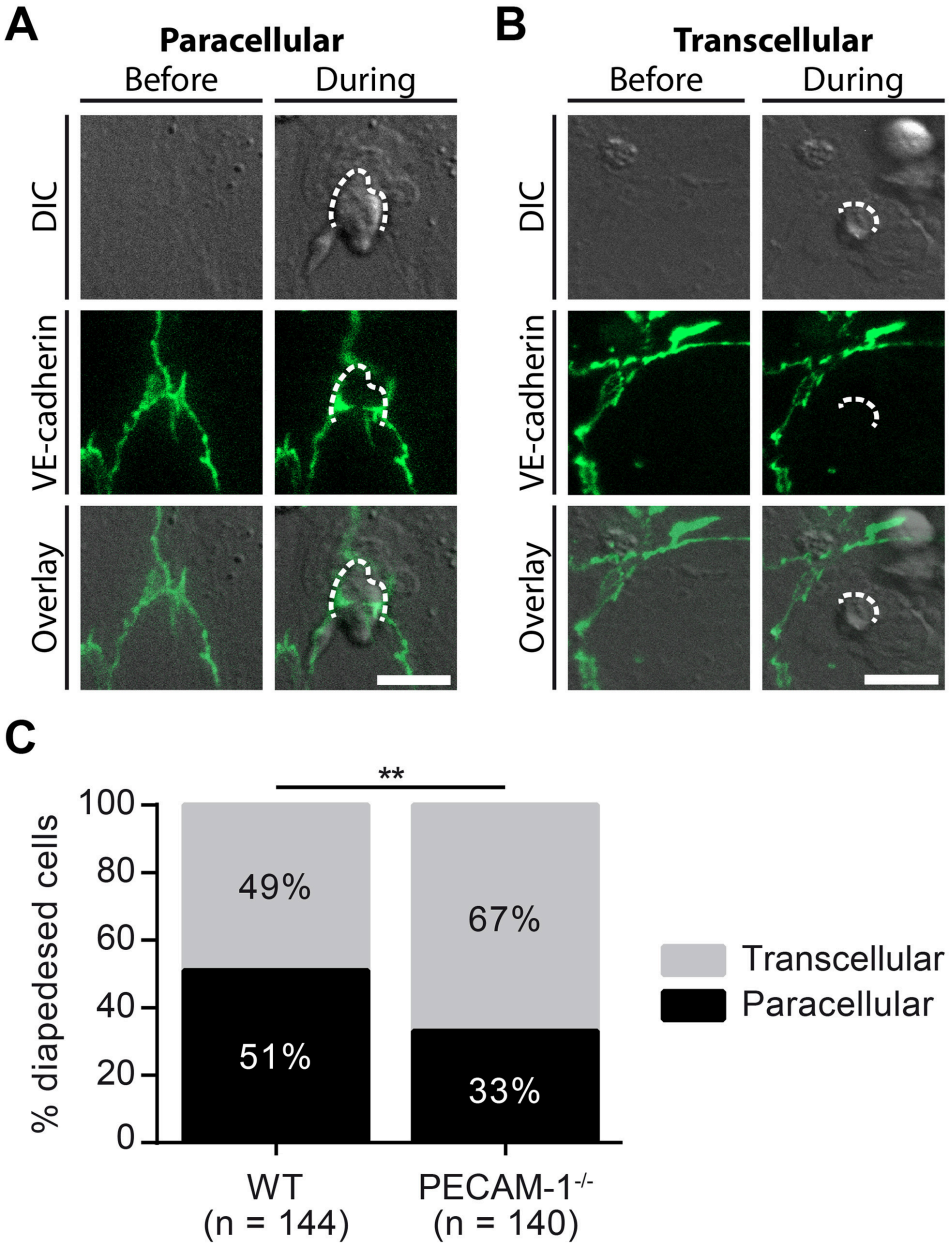
Lack of endothelial PECAM-1 favors transcellular over paracellular diapedesis of encephalitogenic CD4^+^ T cells across pMBMEC monolayers under physiological flow. Confluent WT and PECAM-1^−/−^ VE-CadGFP pMBMEC monolayers were stimulated with IL-1β (20 ng/ml) for 16–24 h prior to live cell imaging. Activated encephalitogenic CD4+ T cells were allowed to arrest on pMBMECs at low flow (0. 1 dyn/cm^2^). After 5 min, the flow was increased to 1.5 dyn/cm^2^. For time-lapse videos, pictures were taken every 30 s for a total of 30 min. For each diapedesis event, the transmigration route was determined as follows: An opening of GFP-expressing endothelial junctions was judged as paracellular diapedesis, while a sustained junctional integrity was indicative of transcellular diapedesis. **(A,B)** Representative pictures of paracellular **(A)** and transcellular **(B)** diapedesis taken before and during T-cell diapedesis. Dashed line, site of transmigration; scale bar, 10 μm; DIC, differential interference contrast. **(C)** In total, 144 and 140 T cells, which completely transmigrated through WT and PECAM-1^−/−^ endothelial monolayers, respectively, recorded in 68 videos (WT, 34 videos; PECAM-1^−/−^, 34 videos) were analyzed. Reported statistics of categorical data result from Pearson's qui-square test. Test statistics: *p* = 0.0016, χ^2^(1) = 9.991, odds ratio = 2.160; ^**^p < 0.01. In order to provide an estimate for data variability, a video-wise representation is provided in Supplementary Figure 2.

Considering the predominant junctional localization of endothelial PECAM-1, we finally asked if the lack of endothelial PECAM-1 affects the cellular pathway of T-cell diapedesis across pMBMEC monolayers. To this end, we crossed the PECAM-1^−/−^ C57BL/6 mice with VE-CadGFP knock-in mice expressing a VE-cadherin-GFP fusion transcript from the endogenous VE-cadherin locus, leading to fluorescent labeling of the endothelial adherens junctions caused by EGFP fused to the VE-cadherin protein (55). Usage of *in vitro* cultured pMBMECs isolated from VE-CadGFP reporter mice, which reportedly do not differ from WT mice e.g., with regard to barrier resistance (12), allows for the visualization of endothelial cell-cell junctions during live cell imaging under physiological flow conditions (Figure 3B) (12, 63). This allowed us to define the cellular pathway of T-cell diapedesis across the pMBMEC monolayers as paracellular, when we observed a transient interruption of the junctional GFP signal (Figure 6A), while those diapedesis events leaving the junctional GFP signal intact were classified as transcellular diapedesis (Figure 6B).

In accordance with our previous findings (12), encephalitogenic T cells crossed the IL-1β-stimulated pMBMEC monolayers equally via the paracellular (51%) and the transcellular (49%) pathway (Figure 6C, Supplementary Figure 2). Unexpectedly, lack of PECAM-1 further increased transcellular T-cell diapedesis across the pMBMEC monolayers to 67%, while only 33% of the T cells crossed PECAM-1^−/−^ VE-CadGFP pMBMEC monolayers via the paracellular route. Thus, lack of endothelial PECAM-1 favors transcellular over paracellular T-cell diapedesis across pMBMEC monolayers.

### Lack of PECAM-1 Does Not Affect Expression of ICAM-1 on pMBMECs

**Figure 7:**
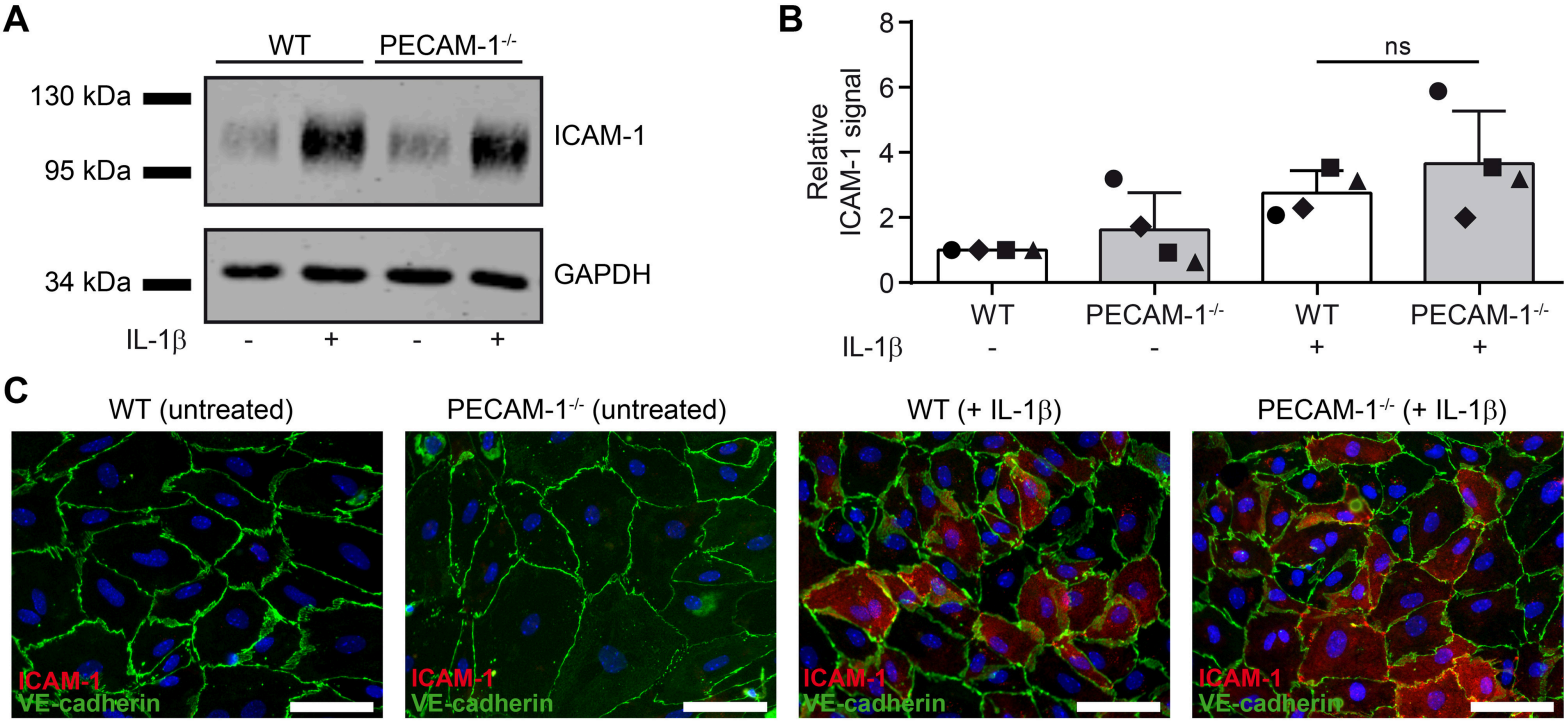
Lack of endothelial PECAM-1 does not affect expression levels of ICAM-1 on pMBMECs. **(A)** WT and PECAM-1^−/−^ pMBMEC monolayers were treated for 16 h either with IL-1β (20 ng/ml) or left unstimulated. Western blots of cell lysates were incubated with anti-ICAM-1 rabbit serum and rabbit anti-GAPDH antibody. One representative experiment out of 4 is shown. **(B)** Quantified ICAM-1 signals were normalized to the reference protein GAPDH. For each experiment, normalized signals were further related to the unstimulated WT sample. Each individual data point is shown and shapes correlate to the respective independent experiments (*n* = 4). Reported statistics result from unpaired, two-tailed Student's *t*-test. Test statistics: *p* = 0.348, *t*(6) = 1.018, *d* = 0.831. Error bar, ± SD; ns, not significant. **(C)** Untreated and IL-1β-stimulated VE-CadGFP endothelial monolayers stained for ICAM-1 expression (rat hybridoma supernatant 25ZC7). Blue, cell nuclei stained with DAPI; Scale bar, 60 μm.

We have previously shown that cell surface levels of endothelial ICAM-1 direct T cells to transcellular vs. paracellular routes of diapedesis across pMBMECs (12). While high levels of endothelial ICAM-1 promoted transcellular T-cell diapedesis, lower ICAM-1 levels favored paracellular T-cell diapedesis across pMBMEC monolayers under physiological flow (12). To exclude that the increased transcellular T-cell diapedesis across PECAM-1^−/−^ pMBMECs was solely due to an inherently altered endothelial ICAM-1 expression on WT vs. PECAM-1^−/−^ pMBMECs, we compared ICAM-1 expression levels in WT and PECAM-1^−/−^ pMBMECs. Western blot analysis of protein lysates from unstimulated or IL-1β-treated WT and PECAM-1^−/−^ pMBMECs (Figures 7A,B; Supplementary Figure 3) showed comparable levels of ICAM-1 protein in WT and PECAM-1^−/−^ pMBMECs under both conditions (Figure 7A). Quantification of Western blot signals showed that ICAM-1 expression did not significantly differ between IL-1β-treated WT and PECAM-1^−/−^ pMBMECs, although ICAM-1 signals varied to a greater extent in PECAM-1-deficient pMBMEC samples (Figure 7B). To study cell surface expression of endothelial ICAM-1 we performed immunofluorescence stainings. We found that ICAM-1 cell surface expression was not uniform but rather varied between individual pMBMECs. However, the ICAM-1 staining pattern was indistinguishable between WT and PECAM-1^−/−^ pMBMEC monolayers (Figure 7C).

Thus, lack of PECAM-1 did not impact on ICAM-1 protein levels and its cell surface distribution on pMBMECs. Therefore, increased transcellular T-cell diapedesis across PECAM-1^−/−^ pMBMECs is not due to increased expression of ICAM-1.

## Discussion

The present analysis of pre-existing microarray data on MS brain lesions (39, 40) highlighted a profound upregulation of *PECAM-1* transcripts in initial white matter as well as active cortical gray matter MS lesions. So far, small-scale gene association studies (64–66) as well as genome-wide association studies (67–69) have not detected any *PECAM-1* gene variants affecting MS susceptibility. However, its strategic localization at BBB interendothelial junctions as well as immunohistochemical findings and blood analyses (33–36, 70) suggest a possible role for PECAM-1 in MS pathogenesis, including immune cell trafficking or regulation of BBB integrity. Here, we could show that in comparison to human control brain tissue, transcript and protein levels of PECAM-1 were upregulated in initial (pre-phagocytic) white matter as well as active cortical gray matter MS lesions. Until now, antibody labeling of post mortem MS and control tissue has solely shown that the constitutive expression of PECAM-1 in blood vessels is maintained during disease (70). However, differential PECAM-1 expression in endothelial cells has not specifically been addressed so far; only perivascular and parenchymal macrophages in active demyelinating lesions have been positively stained for PECAM-1. Western blot quantification of normal appearing white matter, distinct white matter lesions, and normal appearing gray matter in comparison to white and gray matter from healthy control subjects have not yielded any significant differences in PECAM-1 levels (70). However, distinctions between endothelial- and leukocyte-derived PECAM-1 were not possible within protein lysates. Here, we used archival FFPE material obtained from acute and progressive MS cases, of which especially the former present with a high load of inflammation enabling us to examine prominent molecular changes, which would stay undetected if less active disease conditions were used for initial screening. In active white matter lesions from our selected MS brain samples, we detected faint PECAM-1 reactivity on infiltrating immune cells. Endothelial PECAM-1 labeling was quite attenuated compared to initial (pre-phagocytic) white matter lesions, for which the by far strongest protein expression as well as a multiple-fold upregulation of mRNA transcripts was found. Initial (pre-phagocytic) lesions are characterized by commencing disintegration of myelin and microglia activation as well as tissue edema accompanied by a loss of tissue structure and mild axonal injury (45, 46). However, infiltrating lymphocytes or phagocytes are sparse in these lesions. Thus, the increase of *PECAM-1* gene expression in initial (pre-phagocytic) lesions can be attributed to an endothelial origin. It was further suggested that PECAM-1 expression in MS might represent a protective mechanism (70). At least in *in vitro* experiments it was shown that IFN-β, a routinely used disease-modifying drug in MS therapy, significantly increased endothelial PECAM-1 expression (38).

PECAM-1 is a transmembrane protein with six extracellular Ig-like domains (16), which facilitate homophilic (71–73) and heterophilic interactions (74–78). It is expressed in endothelial cell-cell junctions of the vasculature (17) as well as on platelets, neutrophils, monocytes and selected lymphocyte subsets (18–20). Initially, it has been reported that PECAM-1 plays an indispensable role in the transendothelial migration of myeloid cells (21, 79–81). For instance, the transmigration of neutrophils and monocytes through human umbilical vein endothelial cell (HUVEC) monolayers was massively reduced by anti-PECAM-1-antibodies and recombinant PECAM-1 protein (21). In this context, PECAM-1 functions upstream of CD99 and prevents myeloid cells from entering endothelial junctions (21). *In vivo* live cell imaging studies showed that PECAM-1 is neither involved in leukocyte rolling nor in their subsequent arrest on the inflamed endothelium, but rather in the subsequent steps of crossing the vascular wall. In PECAM-1^−/−^ C57BL/6 mice, which do not show alterations in vascular development, blood cell counts, and peripheral organ morphology in naïve conditions (54), leukocytes can still cross the inflamed endothelium in the absence of PECAM-1, but are subsequently trapped at the level of the perivascular basement membrane and are thus inhibited in their migration capacity into the extravascular tissue (54, 82, 83). Other mouse strains such as FVB/n, SJL or the outbred strain Swiss Webster show a more pronounced phenotype in the functional absence of PECAM-1, with clearly reduced leukocyte extravasation under inflammatory conditions (23, 84). Interestingly, in the latter mouse strains, functional or genetic PECAM-1 deficiency was found to arrest leukocytes at the endothelial junctions before diapedesis started (23, 84). These observations underscore that there are still additional components in the diapedesis machinery to be discovered, which are associated with the role of PECAM-1 in leukocyte diapedesis. Indeed, a study has already identified a locus on murine chromosome 2 strongly associated with inflammatory responses in the absence of PECAM-1 (85). Additional puzzling observations come from immune cell-mediated disease models such as experimental autoimmune encephalomyelitis (EAE) (24), collagen-induced arthritis (86, 87), or atherosclerosis in low density lipoprotein receptor (LDLR)- or apolipoprotein E (ApoE)-deficient mice (88, 89), in which absence of PECAM-1 was found to lead to aggravated disease severity.

The majority of studies investigating the role of PECAM-1 in leukocyte diapedesis have focused on monocytes and neutrophils, while the role of endothelial PECAM-1 in T-cell trafficking has rarely been taken into account. Furthermore, there are very few studies addressing the role of PECAM-1 in leukocyte trafficking across the BBB. In fact, one study found the transmigration rate of T cells to be significantly increased across PECAM-1-deficient immortalized mouse brain endothelial cells (24). As endothelial cell lines usually do not possess true BBB-like characteristics, we used in the present study an established *in vitro* BBB model, in which the employed pMBMECs maintain BBB properties (61). This allowed us to investigate the role of PECAM-1 in the maintenance of BBB characteristics as well as in T-cell diapedesis. While absence of PECAM-1 did not affect the overall junctional architecture of pMBMEC monolayers, already under non-inflammatory conditions, lack of PECAM-1 in pMBMECs was associated with a decreased transendothelial electrical resistance (TEER) and increased permeability to small molecular tracers when compared to WT pMBMEC monolayers. These findings are in line with previous data showing that siRNA-silencing of PECAM-1 in endothelial cell lines results in significantly reduced electrical resistances of both human aortic endothelial cells (HAECs) or immortalized human umbilical vein endothelial cells (iHUVECs) (90). In the same study, a potential contribution of PECAM-1 to junctional repair mechanisms was found. After thrombin-induced endothelial barrier disruption, a significantly delayed barrier re-establishment in PECAM-1-silenced endothelial cell lines was reported. Similarly, we have previously shown that *in vitro* histamine challenge of PECAM-1^−/−^ lung endothelial cells leads to a prolonged permeability for Evans Blue diffusion compared to PECAM-1^−/−^ lung endothelial cells transfected with wild-type PECAM-1 (24). Additionally, PECAM-1^−/−^ C57BL/6 mice suffering from EAE showed increased vascular permeability for Evans Blue compared when compared to wild-type mice suffering from EAE (24). Here, we found that while pro-inflammatory IL-1β stimulation further reduced the TEER of PECAM-1^−/−^ pMBMEC monolayers it did not further increase the diffusion of small molecular tracers across PECAM-1^−/−^ pMBMEC monolayers. This may be due to the fact that the diffusion barrier for the small molecular tracers used in the present study across non-stimulated PECAM-1^−/−^ pMBMEC monolayers was already too high, thus prohibiting detection of a further permeability increase by the inflammatory stimulus in the given experimental setting. Taken together, these observations underscore a role for endothelial PECAM-1 in the maintenance of BBB integrity.

To investigate the role of PECAM-1 in T-cell diapedesis across the BBB, we made use of our human *in vitro* BBB model and PECAM-1 blocking antibodies, and found that in contrast to CD99 and ICAM-1, PECAM-1 is not required for human CD4^+^ T cell diapedesis across the BBB. We next used our mouse *in vitro* BBB model and studied the dynamic interaction of *in vitro* activated encephalitogenic CD4^+^ T cells with pMBMECs under physiological flow conditions by live cell imaging. We found that PECAM-1 expression in C57BL/6 mice-derived pMBMECs was dispensable for the arrest and crawling of T cells as well as for the number of T cells crossing the pMBMEC monolayer. However, we observed that endothelial PECAM-1 expression influences the cellular route of T-cell diapedesis. In the absence of endothelial PECAM-1, encephalitogenic CD4^+^ T cells preferentially crossed IL-1β-stimulated pMBMEC monolayers via a transcellular route instead of the typically preferred paracellular route across wild-type pMBMECs.

Lately, we have shown that cell surface levels of endothelial ICAM-1 direct T cells to either transcellular or paracellular routes of diapedesis (12). While low to intermediate ICAM-1 expression on pMBMECs favored paracellular diapedesis of *in vitro* activated CD4^+^ T cells, high endothelial ICAM-1 surface levels led to significantly increased transcellular diapedesis. A similar finding has been described for *in vitro* diapedesis of neutrophils across TNF-α-stimulated iHUVECs overexpressing ICAM-1 (91), where the enhanced surface levels of ICAM-1 on the endothelial cells promoted the transcellular transmigration pathway of polymorphonuclear cells. Translocation of ICAM-1, accompanied by caveolin-1 and F-actin, to the basal plasma membrane and its recruitment to caveolae has been described to initiate the formation of transcellular channels (92). Based on these observations, it could be argued that the described altered T-cell diapedesis route across PECAM-1^−/−^ pMBMECs is triggered by a putative compensatory upregulation of ICAM-1 due to PECAM-1 deficiency. However, our Western Blot analysis revealed no differential expression of endothelial ICAM-1 in the absence of endothelial PECAM-1 on IL-1β-treated pMBMECs. Also, high endothelial ICAM-1 surface expression had been described to reduce crawling distances and crawling speed of encephalitogenic CD4^+^ T cells on pMBMECs (12), which was not observed in the present study.

The cytoplasmic tail of PECAM-1 harbors two immunoreceptor tyrosine-based inhibitory motifs (ITIMs) that, upon phosphorylation, act as docking sites for Src homology 2 (SH2) domain-containing proteins (93–96). Thereby, PECAM-1 can act as inhibitory or stimulatory signal transducer in a cell-specific manner (16, 97). Additionally, PECAM-1 was shown to coordinate the actin cytoskeleton and to associate with β- and γ-catenins (98, 99). Protein kinase C-mediated serine/threonine phosphorylation of PECAM-1 was shown to inhibit its association with γ-catenin, which was then translocated into the nucleus (99). Furthermore, PECAM-1-engagement, in the course of leukocyte transmigration, has been shown to counteract ICAM-1-induced tyrosine phosphorylation of cortactin and cytoskeletal actin rearrangements (100), thus putatively prohibiting excessive endothelial activation and junctional opening (101). BBB junctional molecules provide guidance cues facilitating a strictly controlled paracellular T-cell diapedesis across the BBB e.g., to allow CNS immune surveillance (102). In fact, as we have previously shown, that the majority of T cells crosses pMBMEC monolayers via the paracellular pathway when barrier properties are preserved, while breakdown of junctional integrity in IL-1β-stimulated pMBMECs correlates with increased T-cell diapedesis via the transcellular pathway (12). Thus, loss or incorrect localization of junctional molecules seems to reduce paracellular T-cell diapedesis across the BBB. The BBB is characterized by unique barrier functions, which are, amongst others, maintained by limited vesicular activity (103). The remainder of vesicles comprises caveolin 1-containing caveolae (104, 105). During multiple sclerosis (MS) and EAE, the number of caveolae was found to be increased in the CNS endothelium resulting in clustering of adhesion molecules at the luminal side of the vasculature (92, 106), which in turn might facilitate the transcellular route of T cell diapedesis into the CNS. Indeed, a recent study employing intravital microscopy in claudin-5 reporter mice showed that during the onset of EAE, T-cell diapedesis across the BBB mainly occurs via a caveolae-independent paracellular pathway, while caveolae regulate transcellular T cell diapedesis across the BBB during ongoing neuroinflammation (13).

Taken together, our data demonstrate that PECAM-1 is involved in the regulation of BBB integrity. At the same time, PECAM-1 is neither required for human T-cell diapedesis across human BBB-like endothelial cells *in vitro* nor for murine T-cell diapedesis across pMBMECs under flow *in vitro*. The lack of PECAM-1 on pMBMECs rather directs T-cell diapedesis to the transcellular route, while in its presence T-cell diapedesis across stimulated pMBMECs occurs equally on both the transcellular and the paracellular route. The shift in the preferential T-cell diapedesis pathway across PECAM-1^−/−^ pMBMECs might be due to non-antagonized ICAM-1-signaling in PECAM-1^−/−^ pMBMECs establishing the transcellular pathway via caveolin-1/F-actin-enriched channels (13). ICAM-1 is significantly expressed during early lesion formation in MS and EAE (107). Upregulated vascular PECAM-1 expression in initial (pre-phagocytic) white matter as well as active cortical gray matter MS lesions thus might represent a compensatory mechanism to repair BBB characteristics and to prevent uncontrolled immune cell infiltration via the transcellular pathway, which is seemingly facilitated by inflammatory cytokine (e.g., IL-1β)-mediated ICAM-1 upregulation and ICAM-1 signaling in brain endothelial cells. This hypothesis is further supported by the fact that PECAM-1^−/−^ mice exhibit increased numbers of CNS-infiltrating immune cells in the course of EAE (24). Finally, while studying the functional absence of PECAM-1 at the BBB has allowed us to identify its role in maintaining BBB integrity and in regulating the cellular pathway of T-cell diapedesis across the BBB, overexpression of PECAM-1 may induce additional mechanisms that cannot be predicted from studying absence of PECAM-1. Furthermore, the role of PECAM-1 in mediating the CNS entry of additional immune cell subsets contributing to MS pathogenesis, e.g., CD8^+^ T cells and B cells remains to be explored.

## Author Contributions

IW and ST designed and performed experiments, analyzed data, and wrote the manuscript. HN designed and performed experiments and contributed to manuscript writing. UD managed and maintained the transgenic mouse colonies and edited the manuscript. WM and FG provided reagents, cells and advice for the study and edited the manuscript. FS provided T cells and advice for the study. RL supervised parts of the study and contributed to manuscript writing. HL designed and supervised the analysis of human autopsy tissue and contributed to manuscript writing. BE designed and supervised the *in vitro* studies and wrote and edited the manuscript.

### Conflict of Interest Statement

HL received honoraria for lectures from Novartis, Biogen, and Sanofi Aventis. Moreover, he is a member of advisory boards at Roche and Medday. BE receives research support from Biogen. The remaining authors declare that the research was conducted in the absence of any commercial or financial relationships that could be construed as a potential conflict of interest.
