# Supplementary material for: PECAM-1 Stabilizes Blood-Brain Barrier Integrity and Favors Paracellular T-Cell Diapedesis Across the Blood-Brain Barrier During Neuroinflammation
Source: Front Immunol. 2019 Apr 5;10:711. doi: 10.3389/fimmu.2019.00711 (PMC6460670; doi:10.3389/fimmu.2019.00711)
Supplement: Supplementary file 1 [file Data_Sheet_1.pdf]

Supplementary Material

1 Supplementary Materials and Methods

Flow Cytometry

For the analysis of surface PECAM-1 expression on *in vitro* activated mouse T cells, SJL.PLP4 cells were incubated with 100 µl rat hybridoma supernatants (anti-mouse PECAM-1: clone Mec13.3; isotype control anti-human CD44: clone 9B5) for 30 min. Subsequently, cells were washed twice with FACS-buffer (PBS, 2.5 % FBS, and 0.1 % NaN_3). 1:200 diluted PE-conjugated secondary antibody (Invitrogen) pre-incubated with 10 % mouse serum in FACS-buffer was added to the T cells and incubated for 30 min. After three washes with FACS-buffer, cells were fixed with 1 % PFA in FACS-buffer and flow cytometry was performed using the FACSCalibur (BD Bioscience). Data were analyzed with FlowJo software (FlowJo version 10).

For the analysis of surface expression on human T cell subsets *in vitr*o polarized towards Th1, Th1*, Th2, and Th17, cells were incubated either with directly conjugated APC-Cy7 mouse-anti-human CD31 antibody (5 µl/test, BD Pharmingen, clone WM59) or the respective isotype control (mouse IgG1) for 20 min in FACS-buffer. Subsequently, T cells were washed once with FACS-buffer and flow cytometry was performed using the Attune NxT (Thermo Fisher Scientific) and data were analyzed with FlowJo software.

2 Supplementary Figures

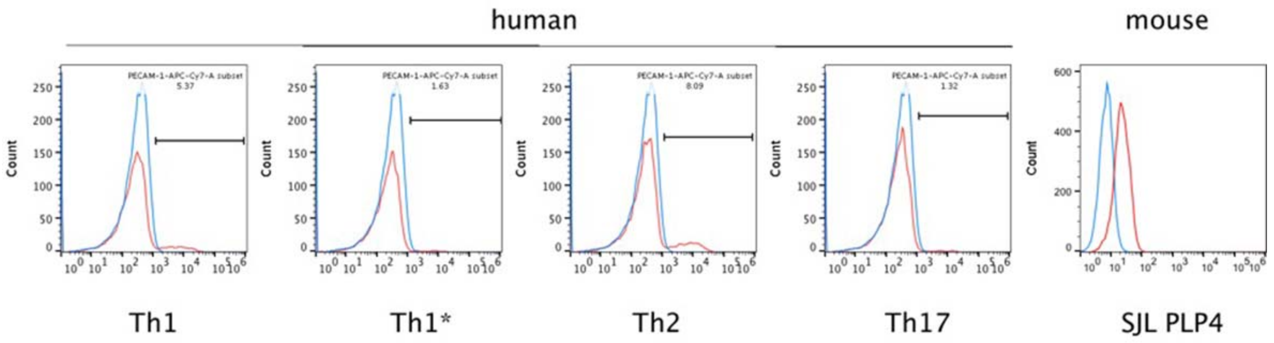

Supplementary Figure 1: PECAM-1 expression on human and mouse T cells

Flow cytometric analysis of PECAM-1 expression on human *in vitro* polarized Th1, Th1*, Th2, and TH17 T cells is shown from one healthy donor. Moreover, flow cytometric analysis of PECAM-1 expression on *in vitro* activated mouse SJL.PLP4 T cells is shown. Histograms show isotype controls (blue line) and PECAM-1 expression (red line).

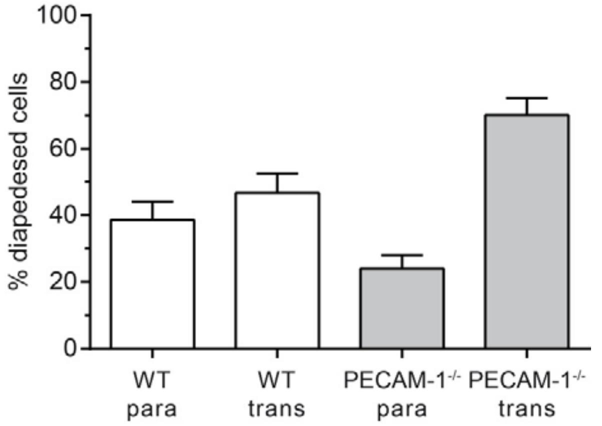

Supplementary Figure 2: Route of diapedesis of encephalitogenic CD4^{+} T cells across pMBMEC monolayers under physiological flow

Data shown in Figure 6C were re-analyzed by calculating percentages of T cells that had crossed pMBMEC monolayers paracellularly (para) or transcellularly (trans) for each individual video. For each genotype, 34 videos were reanalyzed to provide a better estimate of data variability. Altogether, 144 and 140 T cells that completely transmigrated through WT and PECAM-1$^{-/-}$ pMBMEC monolayers, respectively, were included in the analyses. Error bars ± SEM are shown.

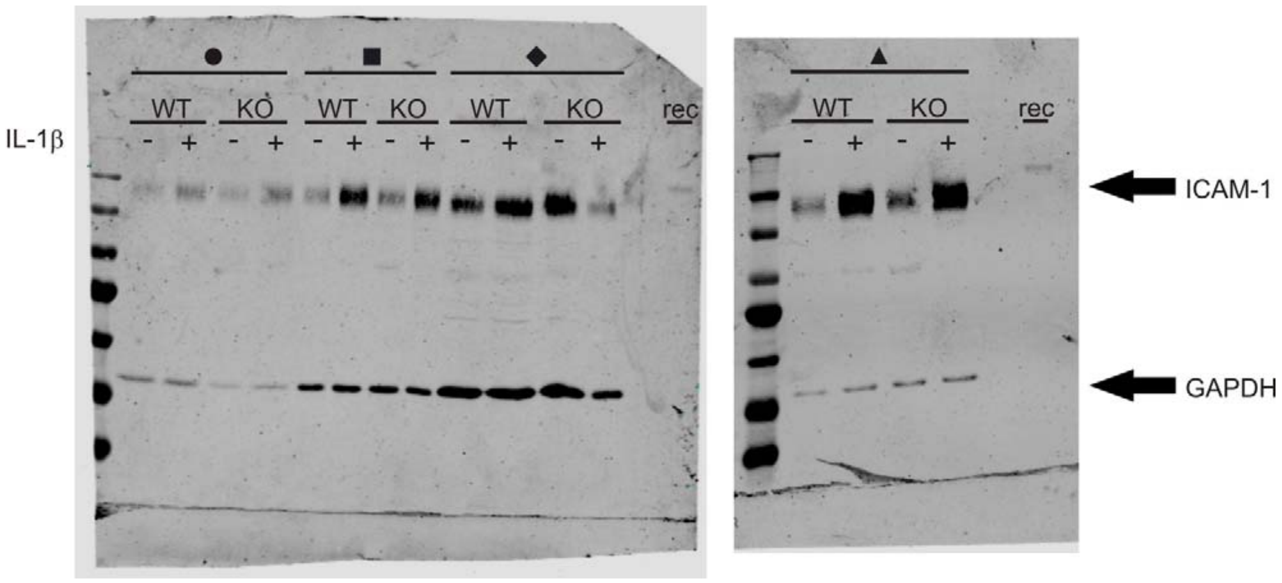

Supplementary Figure 3: Original ICAM-1 Western blots

WT and PECAM-1$^{-/-}$ pMBMEC monolayers were treated for 16 hours either with IL-1β (20 ng/ml) or left unstimulated. Western blots of cell lysates were incubated with anti-ICAM-1 rabbit serum and rabbit anti-GAPDH antibody. Original blots of the four individual experiments are shown. Each experiment's symbol correlates to the symbols used in Figure 7B.
